# Target Trial Emulation in Observational Research—Strengths, Limitations, and Methodological Considerations

**DOI:** 10.1111/jre.70095

**Published:** 2026-03-06

**Authors:** Sebastian‐Edgar Baumeister, Ignacio Leiva‐Escobar, Ryan T. Demmer, Panos N. Papapanou, Thomas Kocher, Birte Holtfreter, Michael Nolde

**Affiliations:** ^1^ Institute of Health Services Research in Dentistry University of Münster Münster Germany; ^2^ Division of Epidemiology, Department of Quantitative Health Sciences Mayo Clinic College of Medicine and Science Rochester Minnesota USA; ^3^ Division of Periodontics, Section of Oral, Diagnostic and Rehabilitation Sciences, College of Dental Medicine Columbia University New York New York USA; ^4^ Department of Restorative Dentistry, Periodontology, and Endodontology University Medicine Greifswald Greifswald Germany

**Keywords:** cohort study, epidemiology, longitudinal study, observational study, study design, target trial emulation

## Abstract

Cohort studies are vulnerable to confounding and time‐related biases. Recent advances emphasize the importance of emulating a hypothetical target trial to minimize several of these biases. The target trial framework is a critical tool for designing cohort studies. It requires explicit specification of protocol components: eligibility criteria, treatment strategies, outcomes, timing of follow‐up (time zero), and the causal contrast to be estimated. Critical to this framework is defining “time zero” when treatment assignment takes place and follow‐up begins, which prevents prevalent user and immortal time bias and ensures proper temporal sequencing of confounders, exposure, and outcome. By guiding design, data collection, and analysis, the target trial framework helps align observational studies with the principles of randomized trials, improving the validity and interpretability of their findings. While it cannot eliminate unmeasured confounding, this approach promotes transparent, bias‐conscious cohort design, minimizes time‐related bias, and supports more robust causal inference with observational data.

## Graphical abstract

The target trial framework is a pragmatic roadmap for observational causal inference that clarifies questions, mitigates time‐related bias, and improves interpretability while acknowledging limits. With transparent protocols, appropriate methods, and suitable data, it can complement randomized trials and inform practice and policy.

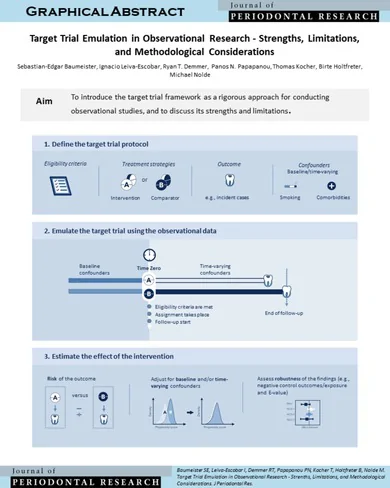

## 1 Introduction

Observational studies serve diverse purposes: they may be descriptive (e.g., estimating the prevalence or secular trends of periodontitis) or predictive (e.g., predicting the first occurrence of disease). However, a primary goal of many observational studies is to assess causal relationships [1]. Because investigators in observational settings do not control the assignment of the exposure, choosing an appropriate design is critical to establishing valid associations and minimizing the biases that frequently plague this research. In this tutorial, we focus on observational studies designed to estimate the effect of a treatment on disease incidence or progression. Specifically, we explore the target trial emulation (TTE) framework [2]. The use of observational studies that seek to explicitly emulate a hypothetical *randomized trial* has received much attention in medical and dental studies in recent years. The idea of posing causal questions in terms of explicit interventions and hypothetical randomized experiments has a long history [3, 4, 5].

Randomized trials are the gold standard for causal inference not only because they use randomization to balance groups, but because they ask precise questions defined by a rigorous protocol. In contrast, observational researchers often overemphasize the lack of randomization while neglecting the equally critical need for a well‐defined research question. This oversight frequently leads to flawed study designs [6]. The TTE framework addresses this by forcing researchers to explicitly specify the protocol of the hypothetical trial they wish to conduct. While TTE cannot eliminate unmeasured confounding—the inherent limitation of observational studies—it effectively prevents other major sources of error, such as *immortal time bias* and *prevalent user bias* [7]. These biases typically arise when researchers fail to synchronize the start of follow‐up (“*time zero*”) with the moment participants meet eligibility criteria and are assigned a treatment strategy.

We introduce the TTE framework as an overarching design principle for cohort studies and provide practical recommendations for its application. We begin by outlining the fundamental sources of systematic error in observational research, including *confounding*, *selection bias*, and *time‐related bias*. We then explain how the TTE approach mitigates these biases by applying the structural rigor of randomized trials to observational data, complemented by statistical methods to adjust for confounding and selection bias. We illustrate the application of TTE using a concrete case study, discuss key implementation challenges, and conclude with a balanced assessment of the framework's strengths and limitations. Key terms (*italicized* throughout the text) are defined in Table 1.

**TABLE 1:** Glossary of terminology.

Active comperator	A design that compares the treatment of interest with an alternative active treatment rather than a “no treatment” or “standard of care” group. This is used to reduce confounding by indication by ensuring the comparator group has similar clinical indications. And it aligns time zero of treated and untreated units
Backdoor path	A non‐causal path flowing from exposure to outcome in a DAG. It typically represents confounding and must be blocked (e.g., by adjustment) to estimate a causal effect
Bias (systematic error)	A systematic deviation of an estimate from the true causal effect, typically resulting from flaws in study design, data collection, or analysis (e.g., confounding, selection bias, or measurement error)
Collider	A variable that is a common effect of two other variables (e.g., exposure and outcome)
Collider bias	Bias introduced by conditioning (selecting, stratifying, or adjusting) on a collider. This artificially creates a non‐causal association between the two causes of the collider, distorting the exposure‐outcome relationship
Confounder	A variable that causes both the exposure and the outcome (or is a proxy for a cause of both). In a DAG, it is a variable that opens a backdoor path
Confounding	Distortion of the estimated causal effect caused by the mixing of the effect of the exposure with the effect of extraneous variables (confounders)
Confounding by indication	A specific type of confounding where the clinical indication for the treatment is also a prognostic factor for the outcome. This makes treated patients inherently different from untreated patients regarding their baseline risk
Depletion of susceptibles	A phenomenon where the most susceptible individuals in a treated group experience the outcome early or stop treatment due to side effects. If a study includes prevalent users, the remaining group is artificially “healthier” or “tolerant,” potentially masking early risks
Directed acyclic graph (DAG)	A visual diagram using nodes (variables) and one‐way arrows (causal links) to represent assumptions about the data‐generating process. Used to identify confounders, colliders, and mediators to guide statistical adjustment
Disjunctive cause criterion	A heuristic for variable selection which suggests adjusting for all observed causes of the exposure, or all observed causes of the outcome, or both, while avoiding adjustment for mediators and instrumental variables
G‐methods	A class of statistical methods (including inverse probability weighting, the g‐formula, and g‐estimation) designed to estimate causal effects. They are necessary to adjust for time‐varying confounding affected by prior treatment, where standard regression methods fail
Immortal time bias	Bias arising when a period of follow‐up during which the outcome cannot occur (by definition) is misclassified as treated time or included in the analysis. This often artificially favors the treatment group
Informative censoring	Censoring (loss to follow‐up) that is related to the outcome of interest. This creates selection bias because the patients who remain in the study are no longer representative of the original population
Instrumental variable	A variable that affects the exposure but has no independent effect on the outcome and shares no common causes with the outcome. Used in specific analyses (IV analysis) to estimate effects in the presence of unmeasured confounding
Intention‐to‐treat effect	The effect of being assigned to a treatment strategy (or initiating it at time zero) regardless of subsequent adherence. In observational studies, this is the effect of treatment initiation
Inverse probability of censoring weighting	A method used to correct for selection bias due to loss to follow‐up. It weights remaining participants to account for those who were censored, creating a pseudo‐population where censoring is random
Inverse probability of treatment weighting	A method used to correct for confounding. It creates a pseudo‐population where treatment assignment is independent of measured confounders by weighting individuals by the inverse of the probability of receiving the treatment they actually received
Mediator	A variable that lies on the causal pathway between the exposure and the outcome. Adjusting for a mediator blocks part of the total causal effect (bias) if the goal is to estimate the total effect
Multivariable regression	A statistical method that estimates the relationship between an exposure and outcome while mathematically holding other measured variables (covariates) constant. Suitable for baseline confounding but often biased for time‐varying confounding affected by prior treatment
*N*ew user (exposure) design	A design that restricts the study population to patients initiating the treatment of interest (excluding those already taking it). This establishes a clear time zero and avoids prevalent user and immortal time bias
New‐user active comparator design	A rigorous design combining the new user design with an active comparator control group. This aligns the start of follow‐up and minimizes confounding by indication
Overadjustment	Bias or loss of precision introduced by adjusting for variables that should not be adjusted for, such as mediators (blocking the effect) or colliders (inducing bias)
Per‐protocol effect	The effect of the treatment if all participants had adhered to the assigned treatment strategy as specified in the protocol. Estimating this in observational studies requires adjustment for post‐baseline confounding
Prevalent user bias	Bias arising from including participants who have been taking the treatment for some time before the study start. These “survivors” may differ from new users because they have tolerated the drug's early side effects
Randomized trial	An experimental study where treatment assignment is determined by chance (randomization). This ensures that treated and control groups are exchangeable at baseline (similar measured and unmeasured characteristics)
Selection bias	Systematic error arising when the selection of participants into the study ‐ or the likelihood of remaining in the study—depends on both the exposure and the outcome (often conceptually described as conditioning on a collider)
Sensitivity	The proportion of true positive cases that are correctly identified by the diagnostic code or definition used in the study
Specificity	The proportion of true negative cases that are correctly identified by the diagnostic code or definition. High specificity is crucial in observational research to avoid biasing relative risks toward the null
Target trial	The hypothetical ideal randomized trial that a researcher would conduct to answer a specific causal question. The observational study attempts to emulate this trial as closely as possible
Time‐related bias	An umbrella term for biases resulting from the misalignment of eligibility, treatment assignment, and the start of follow‐up (time zero), including immortal time and prevalent user bias
Time zero	The synchronized time point in a study when eligibility criteria are met, treatment strategy is assigned, and follow‐up for the outcome begins

## 2 Methodological Challenges in Cohort Studies

To understand the value of the TTE framework, we must first recognize the common methodological pitfalls that produce erroneous findings in standard cohort studies. In the following sections, we describe these biases before outlining how TTE design principles, implementation steps, and analysis strategies systematically address them.

### 2.1 Confounding and Selection Bias

In cohort studies evaluating treatment effects, confounding and selection bias are major challenges. Consider the case study in this paper, which assesses whether thiazides—a common antihypertensive medication—increase the risk of dental caries. We compared users initiating thiazide monotherapy to those initiating non‐thiazide monotherapy [8]. Confounding occurs when a variable influences both the treatment assignment and the outcome, thereby creating a non‐causal association. For example, obesity is a cause of both antihypertensive choice and caries. Without adjustment, a comparison of caries incidence would be biased by the uneven distribution of obesity between groups. This can be visualized using a *Directed Acyclic Graph* (DAG) (Figure 1A), where confounding is represented by an open “backdoor path” from the outcome to the treatment [9]. DAGs are powerful tools for explicitly mapping causal assumptions and identifying the necessary variables to close these backdoor paths.

**FIGURE 1:**
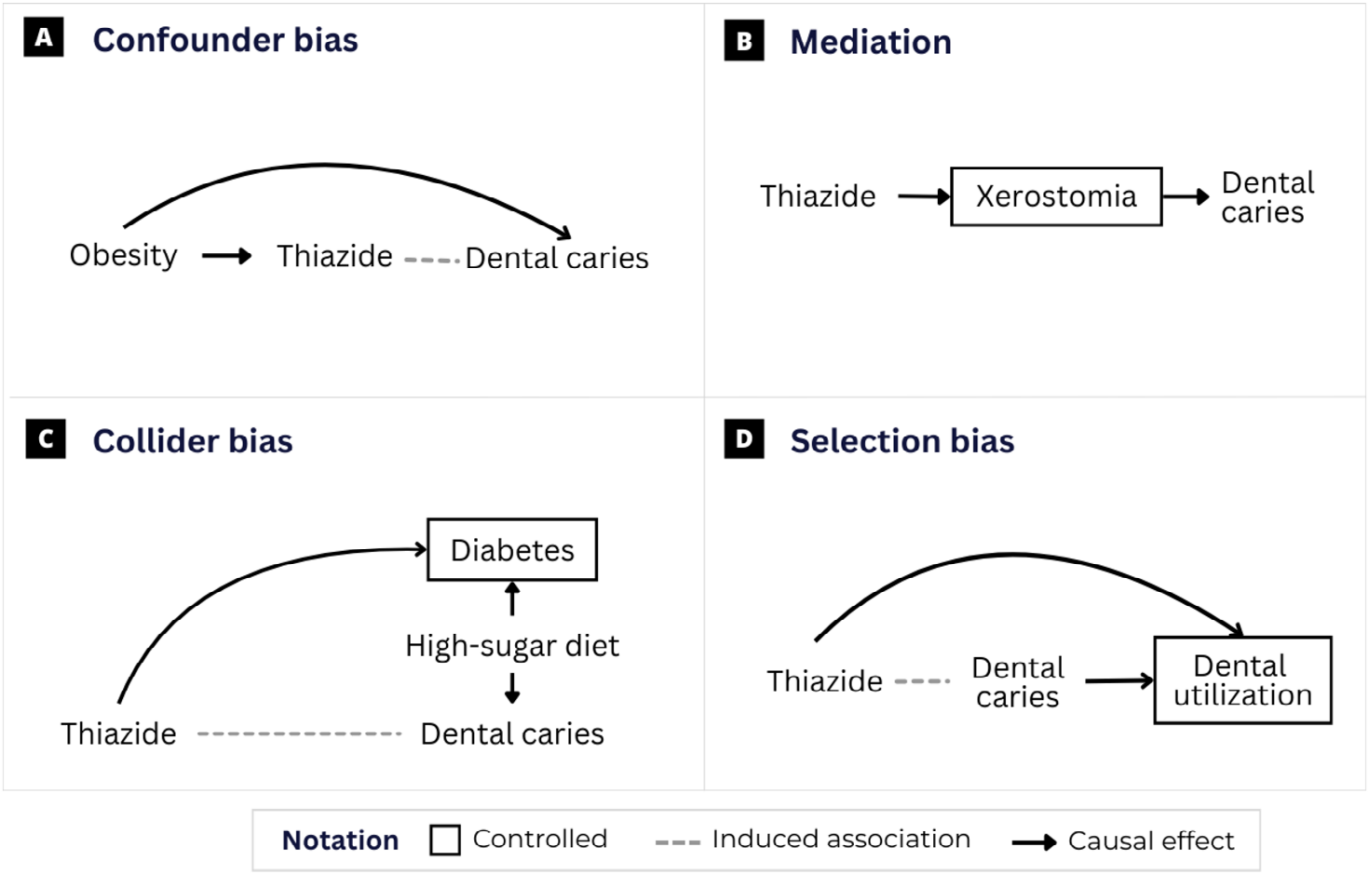
Confounding, mediation and selection bias illustrated using causal diagrams.

However, incorrect adjustment can induce bias. One form is *overadjustment*, which occurs when researchers adjust for a *mediator* on the causal path between treatment and outcome. For instance, xerostomia (dry mouth) is a mechanism by which thiazides may cause caries. Adjusting for xerostomia blocks this path, obscuring the total effect of the drug (Figure 1B). Another form of bias is *collider bias*. A collider is a variable influenced by two other variables (e.g., treatment and outcome). In a DAG, a path containing a collider is naturally blocked. However, conditioning on the collider—either by statistical adjustment or by selecting participants based on it—unblocks the path, creating a spurious association. For example, if thiazide use increases the risk of diabetes, and a high‐sugar diet also increases diabetes risk, then diabetes is a collider (Figure 1C). Adjusting for diabetes would induce a non‐causal association between thiazides and diet (and subsequently caries). Similarly, selecting participants based on a collider introduces selection bias. In our example, if we restricted the analysis only to patients who visit the dentist regularly, we might introduce bias. Thiazides cause dry mouth, which prompts dental visits; caries also prompts dental visits. Conditioning on dental visits (the collider) creates a false association between thiazides and caries even if none exists (Figure 1D).

Selecting the right confounders is therefore critical. The general principle is to adjust for covariates that close backdoor paths without blocking causal paths (mediators) or opening collider paths. Ideally, confounders should be measured temporally prior to the exposure. When the full causal structure is unknown, researchers can apply the *disjunctive cause criterion*: adjust for all pre‐exposure covariates that are causes of the treatment, the outcome, or both, while avoiding *instrumental variables* [10].

### 2.2 Time‐Related Bias: Immortal Time and Prevalent Users

A second major challenge involves the misalignment of time scales, leading to immortal time bias and prevalent user bias. Immortal time bias arises when there is a period of follow‐up during which the outcome cannot occur by design, yet this time is credited to the treatment group [11, 12]. For example, if patients are classified as “users” based on a prescription filled months after the study starts, the time between baseline and that prescription is “immortal” (Figure 2A). Because the patient had to survive event‐free to eventually fill the prescription, this period artificially lowers the event rate in the treated group, biasing results in favor of the treatment. Conversely, prevalent user bias occurs when a study includes individuals who have already been taking the medication for some time before baseline. This design inherently selects “survivors” of the early treatment period [13]. This relates to the *depletion of susceptibles*: high‐risk individuals—those susceptible to early side effects or adverse events—may have stopped treatment or experienced the outcome before the study began. The remaining prevalent users effectively represent a “healthier” sub‐population that tolerates the drug well. As shown in Figure 2B, counting only new caries after baseline excludes patients (D and E) who developed caries early in their treatment course. Furthermore, because treatment started in the past, baseline confounders are measured after the decision to treat has been made, making it difficult to adjust for the patient's true prognostic profile [14]. A classic example is the discrepancy in hormone replacement therapy studies: prevalent user designs suggested a protective effect on heart disease, which was later disproven by randomized trials and new‐user observational designs [15].

**FIGURE 2:**
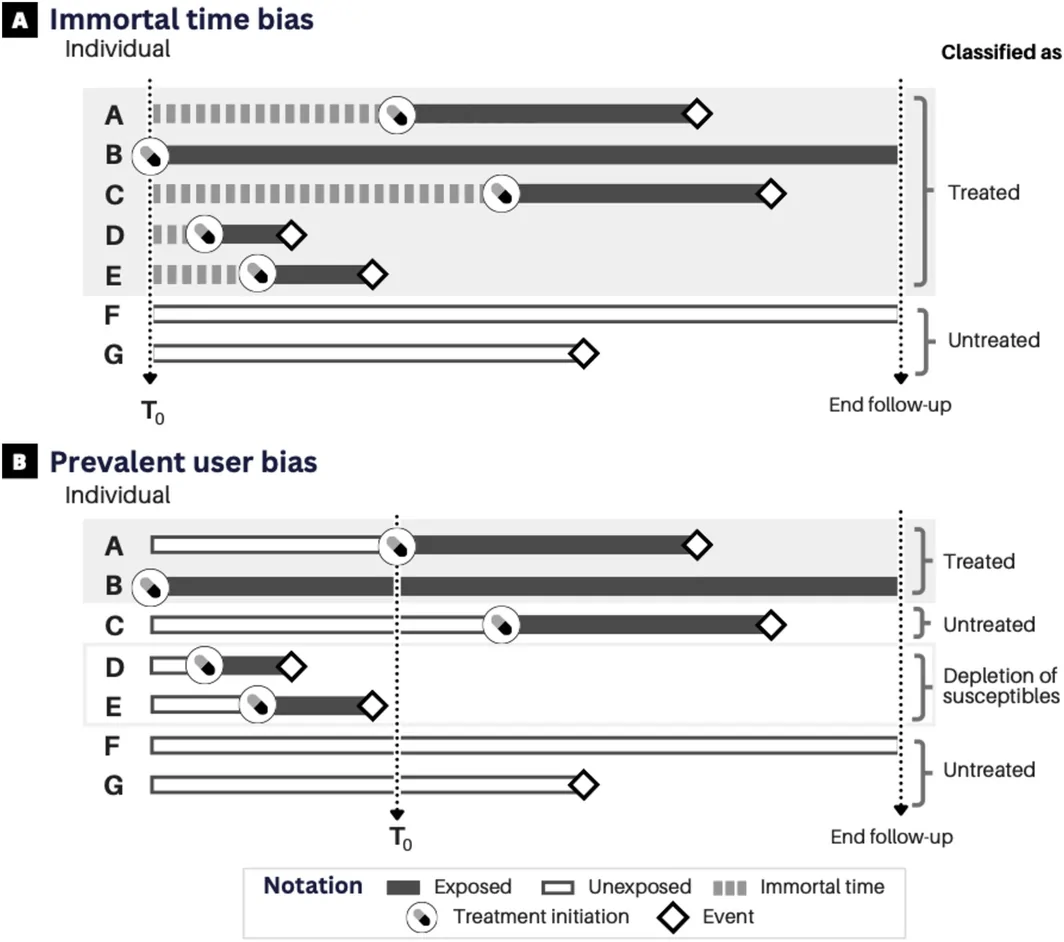
Depiction of immortal time and prevalent user bias.

Both biases are best avoided by using a new user design, which restricts analysis to individuals initiating the exposure. By aligning the start of follow‐up with the start of exposure (time zero), researchers ensure that all downstream events are captured and that “immortal” time is eliminated [11, 16]. This principle applies equally to medical treatments, behavioral interventions (e.g., smoking cessation), and policy changes [17, 18, 19, 20, 21].

## 3 Overview of the Target Trial Emulation Framework

Over the past two decades, methodologies for minimizing bias in observational research have advanced significantly. The TTE framework represents a culmination of these advances, applying the rigorous design principles of randomized trials to observational data. TTE involves designing a hypothetical randomized trial—the “target trial”—that would ideally answer the causal question, and then emulating its key components as closely as possible using observational data. Hernán and Robins [22] codified this process into two distinct steps: (1) specifying the protocol of the hypothetical target trial, and (2) explicitly emulating each protocol component (eligibility, treatment strategy, assignment, follow‐up, outcome, causal contrast, and analysis plan) using the available data. This structured approach forces researchers to define the causal question precisely before analysis begins. While observational data often prevent perfect emulation of every component (e.g., randomization is impossible), this process makes deviations explicit and manageable. To guide this process, researchers can utilize tools like CERBOT [23] and report their findings using the TARGET Statement [24]. In the following sections, we outline these key protocol elements, highlighting essential principles and illustrating their application in our case study.

### 3.1 Eligibility Criteria

Eligibility criteria define the study population and determine the generalizability of the results. In a randomized trial, criteria such as age, comorbidities, and diagnostic history must be met before randomization. TTE must mirror this: all eligibility criteria must be satisfied prior to “time zero” (baseline). Using information from the future (e.g., “patients who survived 6 months”) introduces selection and immortal time biases.

To mimic the active engagement seen in trials, TTE often requires criteria like regular prior healthcare contact [6]. Additionally, a “run‐in” period (e.g., 180 or 365 days prior to baseline) is typically used to exclude prevalent outcomes and exposures and to assess comorbidities and other covariates. If observational data lack specific variables found in a trial (e.g., specific lab values), researchers must modify the target trial protocol to reflect what is feasible, reporting these modifications transparently using a CONSORT‐style flow diagram.

Case Study: We aligned our eligibility criteria closely with a hypothetical trial (Table 2), including individuals ≥ 18 years with a hypertension diagnosis. We excluded those with prior antihypertensive use or recent caries diagnosis (past 180 days) to ensure a “new user” cohort free of the outcome at baseline.

**TABLE 2:** Protocol of a target trial and emulation using All of US data.

Protocol components	Target trial specification	Emulation
Eligibility criteria	Inclusion criteria: People aged ≥ 18 years with a diagnosis of hypertension Exclusion criteria: Antihypertensive use in the 180 days before randomization Dental caries diagnosis within 180 days before randomization Patients with diagnosis of secondary hypertension Patients with hypertension related to pregnancy	Inclusion criteria: People aged ≥ 18 years with a diagnosis of hypertension Minimum record history of 365 days before the first observed antihypertensive prescription (i.e., treatment initiation) Exclusion criteria: Antihypertensive use in the 180 days before treatment initiation Recorded dental caries within 180 days before treatment initiation Patients with diagnosis of secondary hypertension Patients with hypertension related to pregnancy
Treatment strategies	Patients randomly assigned to: Initiate treatment with thiazide monotherapy at any doses Initiate treatment with an angiotensin II receptor blocker, an angiotensin‐converting enzyme inhibitor, a calcium channel blocker, or a beta‐blocker at any dose	Same as the target trial, but initiation was identified as the first prescription between January 1, 1990, and December 31, 2017
Treatment assignment	Eligible individuals are randomly assigned to one of the strategies. Individuals are aware of the strategy they are assigned to	Individuals are randomly assigned within levels of baseline covariates: Age, gender, ethnicity, region, year of prescription, polypharmacy, xerostomia‐inducing drug, prior hospitalization/dental visit, Charlson comorbidity index
Primary endpoint	Incident dental caries	Same as target trial
Follow‐up period	For each individual, follow‐up starts at the time of assignment to a strategy and ends at incidence of dental caries, death, loss to follow‐up, 5 years, or end of the study, whichever comes first	Same as the trial. The end of the study was 31 December 2019
Causal contrast	Intention‐to‐treat effect and per‐protocol effect	Observational analogue of the intention‐to‐treat effect
Statistical analysis	Intention‐to‐treat analysis: estimate cumulative risk of dental caries under each strategy and risk differences with 95% confidence interval	Same as the intention to treat analysis. Additionally, using inverse probability weighting to adjust for confounding and censoring

### 3.2 Treatment Strategies

The protocol must specify exactly what participants are assigned to receive (drug, dose, frequency). Researchers must choose between emulating an initiation strategy (assessing the effect of starting treatment, regardless of adherence) or a sustained strategy (assessing the effect of adhering to treatment over time). Constraints of observational data mean that placebo controls and blinding cannot be emulated; thus, TTE resembles an open‐label pragmatic trial [25].

Case Study: We emulated an initiation strategy comparing: (1) initiation of thiazide monotherapy versus (2) initiation of non‐thiazide monotherapy. Both strategies allowed for any dose or frequency.

### 3.3 Treatment Assignment

In a target trial, randomization ensures exchangeability between groups. In an emulation, treatment assignment is determined by clinical practice. Therefore, we “assign” participants to the strategy consistent with their observed data at baseline. To mimic randomization, we must adjust for baseline covariates under the assumption of conditional exchangeability (no unmeasured confounding).

#### 3.3.1 Case Study

We assigned participants based on their first prescription. We assumed measuring age, gender, ethnicity, region, prescription year, polypharmacy, xerostomia‐inducing drugs, and comorbidities was sufficient to control for confounding.

### 3.4 Outcomes

Outcomes must be pre‐specified. Because emulations rely on secondary data rather than adjudicated clinical measures, researchers typically use validated diagnostic codes [26]. When selecting codes, the heuristic is to prioritize *specificity* over *sensitivity* [27]. Low specificity (false positives) biases relative risks toward the null, whereas low sensitivity (false negatives) primarily reduces precision without introducing substantial bias.

#### 3.4.1 Case Study

Our outcome was incident dental caries, operationalized by identifying specific diagnostic codes in dental records.

### 3.5 Follow‐Up

In a target trial, “time zero” is the synchronized moment when eligibility is met, treatment is assigned, and outcome counting begins. TTE must rigorously replicate this synchronization. Misalignment—such as starting follow‐up after treatment initiation—introduces immortal time bias [6]. Follow‐up ends at the event, death, or censoring. If *censoring* is “*informative*” (related to the outcome), it introduces selection bias, which may require statistical adjustment (e.g., *inverse probability of censoring weighting*).

#### 3.5.1 Case Study

Time zero was the date of the first relevant prescription. We ensured eligibility was met at this date. Follow‐up continued until caries diagnosis, death, loss to follow‐up (defined by the last healthcare encounter), or the administrative end of study (5 years).

### 3.6 Causal Contrasts

Two contrasts are standard: the *intention‐to‐treat* (ITT) effect and the *per‐protocol* (PP) effect. The ITT effect (effect of assignment) is robust in randomized trials but requires baseline adjustment in TTE. The PP effect (effect of adherence) is harder to estimate; in both trials and TTE, it requires censoring and reweighting non‐adherent participants and adjustment for time‐varying confounding [9].

#### 3.6.1 Case Study

We estimated the observational ITT effect: “The difference in 5‐year caries risk if all eligible adults initiated thiazide vs. non‐thiazide monotherapy.” This required adjustment only for baseline confounders.

### 3.7 Statistical Analysis

The choice of method depends on the causal contrast. For ITT analyses, baseline adjustment via *multivariable regression* or propensity score matching is often sufficient. However, for PP analyses involving time‐varying treatments or censoring, standard regression fails if confounders are affected by prior treatment. In these cases, *g‐methods* (like inverse probability weighting) are necessary to correctly adjust for time‐varying confounding.

#### 3.7.1 Case Study

We used *inverse probability of treatment weighting* to adjust for baseline confounders and estimated the risk difference for the ITT contrast.

## 4 How Target Trial Emulation Avoids Time‐Related Bias

A fundamental requirement of TTE is the synchronization of eligibility, treatment assignment, and the start of follow‐up (time zero). Failure to align these elements introduces prevalent user and immortal time biases [7]. Defining time zero is particularly challenging when comparing treatment initiation to “no initiation” (non‐users). Because non‐users have no “index event” (like a prescription), there is no obvious start date. Comparing treated patients to all non‐users inadvertently credits the non‐users with immortal time. Strategies to solve this include:

- Risk‐set (incidence density) sampling: Matching each initiator at time *t* to an eligible non‐initiator at time *t*, assigning them the same start date [12].
- Sequential nested trials: Emulating a new trial at each time point (e.g., every month) where eligibility is met, then pooling the results [6].
- Clone‐censor‐weight: Participants are “cloned” into both arms, censored when their data deviates from the assigned protocol, and weighted to handle informative censoring [28].

### 4.1 Case Study

We avoided these complexities by using an active comparator design. By comparing thiazide initiators to non‐thiazide initiators, time zero was clearly defined for everyone as the date of the first prescription, eliminating time‐related bias by design [11, 13, 29].

## 5 How Target Trial Emulation Minimizes Confounding and Selection Bias

### 5.1 Addressing Confounding by Design: The Active Comparator

Since TTE lacks randomization, causal identification relies on the untestable assumption of conditional exchangeability (no unmeasured confounding). While DAGs help identify confounders, design choices are the first line of defense. The most effective strategy is the active comparator design [11, 13]. Comparing treated patients to non‐users often yields bias because non‐users differ in health‐seeking behavior. Comparing the target drug to another active drug prescribed for the same indication (e.g., thiazides vs. non‐thiazides for hypertension) ensures groups are more exchangeable regarding unmeasured health factors.

### 5.2 Statistical Adjustment: Inverse Probability Weighting

While design reduces confounding, statistical adjustment removes remaining imbalances. For complex scenarios involving time‐varying treatments or informative censoring, g‐methods like inverse probability weighting are essential [9].

- Inverse probability of treatment weighting: Weights participants by the inverse probability of receiving their observed treatment. This creates a pseudo‐population where treatment is independent of measured confounders.
- Inverse probability of censoring weighting: Weights participants by the inverse probability of remaining in the study, correcting for selection bias caused by informative loss to follow‐up.

When combined, these weights allow for unbiased estimation provided the assumptions of no unmeasured confounding, positivity, and correct model specification are met.

### 5.3 Assessing Robustness: Bias Analysis

Because unmeasured confounding is always a risk, sensitivity analyses are crucial. Quantitative bias analysis—such as the E‐value—gauges how strong an unmeasured confounder would need to be to explain away the observed effect [30]. Triangulation with negative controls (outcomes not plausibly affected by the treatment but sharing a similar bias structure) provides a falsification test; non‐null associations suggest residual bias. Additional design‐based probes include varying run‐in and washout periods, redefining outcomes, adjusting grace periods, and altering confounder sets [31].

## 6 Conclusions: Strengths, Limitations and Methodological Considerations

Observational research has long been criticized for producing inconsistent or misleading results. Historically, much of this failure was attributed to residual confounding. However, re‐analyses of major discrepancies between observational studies and randomized trials—such as those involving hormone replacement therapy or statins—reveal that the culprit was often not unmeasured confounding, but rather “researcher‐inflicted” design errors [15, 32]. Specifically, the common practice of identifying participants based on current treatment status creates cohorts of prevalent users who have “survived” early treatment effects. This leads to the depletion of susceptibles and immortal time bias, fundamentally skewing risk estimates.

The TTE framework offers a corrective lens. By forcing researchers to explicitly define and emulate a hypothetical randomized trial, the framework mandates the synchronization of eligibility, treatment assignment, and the start of follow‐up at a precise “time zero.” This rigorous alignment minimizes time‐related biases and ensures a clear temporal sequence where confounders are measured prior to intervention. Importantly, this logic extends far beyond pharmacoepidemiologic research. The TTE framework has been successfully applied to evaluate complex public health interventions, including physical activity, smoking cessation, housing policies, and political reforms [16, 17, 18, 19, 20, 21]. It compels researchers to move from searching for associations to defining precise causal questions with high clinical or policy relevance. Table 3 summarizes strengths and limitations of the TTE framework.

**TABLE 3:** Strength and limitations of the target trial emulation framework.

Feature	Strengths	Limitations
Study design & protocol	Clarifies the research question: Forces researchers to explicitly define the causal contrast (eligibility, treatment, outcome) before analysis, preventing “data dredging” Prevents self‐inflicted biases: Rigorous synchronization of eligibility, treatment assignment, and follow‐up (time zero) eliminates immortal time bias and prevalent user bias.	Complexity: Requires extensive domain knowledge and epidemiological expertise to correctly map the ideal trial protocol to available observational data Feasibility: Not all questions can be emulated; if the data cannot support the protocol (e.g., inability to define a clear time zero), the study must be abandoned or the question modified
Causal inference	Explicit assumptions: Makes the assumptions required for causal inference (e.g., conditional exchangeability, positivity) explicit and transparent Design‐based control: Encourages the use of active comparator designs to minimize confounding by indication	Lack of randomization: Cannot eliminate unmeasured confounding. Validity relies entirely on the untestable assumption that all relevant confounders are measured and adjusted for No blinding or placebo: Cannot emulate placebo‐controlled trials or blinding; effectively restricted to emulating open‐label pragmatic trials
Data & generalizability	Real‐world evidence: Utilizing observational data allows for the evaluation of treatment effects in broader, more representative populations than typical randomize trials (higher external validity) Large sample sizes: Enables the study of rare outcomes or long‐term effects that are often unfeasible in randomized trials due to cost or logistics	Data quality: Results are limited by the granularity of available data (e.g., lack of clinical lab values, poor capture of lifestyle factors like smoking) Outcome misclassification: Reliance on diagnostic codes rather than adjudicated clinical outcomes may reduce precision
Statistical analysis	Flexibility: Compatible with advanced statistical methods (like g‐methods) to handle complex scenarios, such as time‐varying confounding and informative censoring	Statistical difficulty: Emulating per‐protocol effects or adjusting for time‐varying confounding requires sophisticated statistical programming that may be beyond standard curriculum
Ethics	Ethical Feasibility: Allows for the investigation of harmful exposures (e.g., smoking) or interventions where randomization would be unethical	Results must be interpreted with caution (unmeasured bias) to avoid misleading clinical recommendations based on biased observational data

Implementing a target trial emulation is rarely a linear process; it is an iterative dialog between the research question and the available data. Investigators must first specify the “ideal” target trial—the perfect experiment they would conduct with unlimited resources. They must then attempt to map this protocol to their observational dataset. This process often exposes critical gaps: the temporal granularity of records may be insufficient to define time zero, or data on key confounders may be missing.

This friction between the ideal and the realistic is a feature of the framework, not a bug. It necessitates deep collaboration between clinicians and epidemiologists. If the data cannot support the emulation of essential protocol components, the question must be modified, or the study abandoned. This “fail fast” mechanism prevents the generation of low‐quality evidence. Conversely, if emulation is feasible, the protocol should be registered prior to analysis to ensure transparency. Merely labeling a study as a ‘target trial’ does not validate it; validity comes from strict adherence to the principles of trial emulation [33].

Despite its structural rigor, the TTE framework is not a panacea. It addresses design‐based biases (selection and time), but the fundamental limitation of observational research remains: the lack of randomization. TTE cannot guarantee the absence of unmeasured confounding [33]. Sophisticated emulation techniques cannot compensate for poor‐quality data. The success of TTE relies heavily on the quality of the underlying data. While fields like cardiology or oncology benefit from massive repositories like the UK Biobank or the All of US Program. The data landscape in dentistry presents unique challenges. High‐quality, longitudinal dental data linked to systemic health records remain scarce.

To fully leverage the TTE framework, the dental research community must advocate for and construct integrated data infrastructures [34]. Future success depends on accessing longitudinal cohorts with frequent, time‐stamped re‐measurements of oral health status or integrated routine data that links dental claims (procedures) with medical claims (diagnoses/medications) and electronic health records (e.g., periodontal charts). Ideally, large administrative databases should be supplemented with primary data collection or “deep phenotyping” in sub‐populations to capture granular confounders often missing in billing codes, such as smoking status, specific oral hygiene behaviors, or socioeconomic determinants [34]. By improving data infrastructure and adopting rigorous design principles, dental researchers can move from simple association studies to robust causal inferences that shape clinical guidelines.

In sum, the target trial framework is a pragmatic, design‐based roadmap for observational causal inference. It elevates question formulation, tames time‐related biases, and improves interpretability, while openly acknowledging the limits of nonrandomized data. When paired with transparent protocols, appropriate causal methods, rigorous diagnostics, and fit‐for‐purpose data, TTE can yield evidence that complements randomized trials and better informs clinical practice and public policy.

## Funding

This study was supported by the Deutsche Forschungsgemeinschaft (DFG, grant number 552087827). Open Access funding was enabled and organized by Projekt DEAL.

## Conflicts of Interest

The authors declare no conflicts of interest.

## Data Availability

Data sharing not applicable to this article as no datasets were generated or analyzed during the current study.
